# The development and expression of physical nicotine dependence corresponds to structural and functional alterations in the anterior cingulate-precuneus pathway

**DOI:** 10.1002/brb3.227

**Published:** 2014-03-17

**Authors:** Wei Huang, Jean A King, W W Sanouri Ursprung, Shaokuan Zheng, Nanyin Zhang, David N Kennedy, Douglas Ziedonis, Joseph R DiFranza

**Affiliations:** 1 Department of Psychiatry, University of Massachusetts Medical School55 Lake Avenue North, Worcester, Massachusetts, 01655; 2 Department of Family Medicine and Community Health, University of Massachusetts Medical School55 Lake Avenue North, Worcester, Massachusetts, 01655; 3 Department of Radiology, University of Massachusetts Medical School55 Lake Avenue North, Worcester, Massachusetts, 01605

**Keywords:** Addiction, nicotine, resting-state functional connectivity

## Abstract

**Introduction:**

Perturbations in neural function provoked by a drug are thought to induce neural adaptations, which, in the absence of the drug, give rise to withdrawal symptoms. Previously published structural data from this study indicated that the progressive development of physical dependence is associated with increasing density of white matter tracts between the anterior cingulum bundle and the precuneus.

**Methods:**

Using functional magnetic resonance imaging, we compared 11 smokers after 11 h of abstinence from nicotine and after satiation, with 10 nonsmoking controls, using independent component analysis for brain network comparisons as well as a whole brain resting-state functional connectivity analysis using the anterior cingulate cortex as a seed.

**Results:**

Independent component analysis demonstrated increased functional connectivity in brain networks such as the default mode network associated with the withdrawal state in multiple brain regions. In seed-based analysis, smokers in the withdrawal state showed stronger functional connectivity than nonsmoking controls between the anterior cingulate cortex and the precuneus, caudate, putamen, and frontal cortex (*P* < 0.05). Among smokers, compared to the satiated state, nicotine withdrawal was associated with increased connectivity between the anterior cingulate cortex and the precuneus, insula, orbital frontal gyrus, superior frontal gyrus, posterior cingulate cortex, superior temporal, and inferior temporal lobe (*P* < 0.02). The intensity of withdrawal-induced craving correlated with the strength of connectivity between the anterior cingulate cortex and the precuneus, insula, caudate, putamen, middle cingulate gyrus, and precentral gyrus (*r* = 0.60–0.76; *P* < 0.05).

**Conclusions:**

In concordance with our previous report that structural neural connectivity between the anterior cingulate area and the precuneus increased in proportion to the progression of physical dependence, resting-state functional connectivity in this pathway increases during nicotine withdrawal in correlation with the intensity of withdrawal-induced craving. These findings suggest that smoking triggers structural and functional neural adaptations in the brain that support withdrawal-induced craving.

## Introduction

Although the symptoms of nicotine withdrawal are primarily psychological, convention holds that drug withdrawal syndromes indicate physical dependence (PD). Perturbations in neural function provoked by a drug are thought to induce counter-posing homeostatic neural adaptations, which, in the absence of the drug, give rise to withdrawal symptoms. This model assumes that with physical dependence, homeostatic neural adaptations are always present in a latent state, and become manifest in a dynamic state during drug withdrawal. The purpose of this study is to identify neural correlates of the latent state of PD to nicotine, and the dynamic state of nicotine withdrawal.

Craving is an important symptom of PD that manifests dynamically during withdrawal. (American Psychiatric Association 2000) Withdrawal-induced craving (WIC) is that which is triggered by abstinence and relieved by the administration of nicotine. WIC is triggered through different mechanisms than other forms of craving, such as cue-induced craving. Smokers describe three qualitatively distinct forms of WIC of increasing severity, and as PD develops, these three forms of WIC are experienced in the same set order. (DiFranza et al. 2010, 2011, 2012b) The sequential appearance of the three forms of WIC tracks the progressive development of PD and provides a way to measure it. A validated survey measure of the Level of PD allows a smoker's progress along this continuous developmental sequence to be determined in >99% of cases. (DiFranza et al. 2011) The fact that the three forms of WIC develop in the same sequence in all smokers suggests that the neural changes responsible for the latent state of PD might also develop in the same sequence in all smokers. This suggests that homeostatic neural adaptations that underlie PD might be identified by correlating the Level of PD with alterations in neural structure.

Indeed, analyses of the diffusion tensor imaging (DTI) data from part of this study (reported elsewhere) identified an inverse correlation (*r* = −0.68) between the Level of PD and fractional anisotropy (FA, a measure of white matter organization) in the left anterior cingulum bundle (ACb). (Huang et al. 2013) FA in this area also correlated with scores on the Hooked on Nicotine Checklist (HONC), another measure of nicotine addiction. DTI indicated that progression along the Levels of PD corresponds with increased density of white matter tracts between the ACb and the precuneus (*r* = 0.75, *P* < 0.05), but decreased density of white matter tracts between the ACb and the white matter approaching the frontal cortex (*r* = −0.86, *P* < 0.001). (Huang et al. 2013) As these analyses suggested a role for the ACb in the development of PD, and whereas WIC is the dynamic manifestation of PD, we sought to examine the role of the anterior cingulate cortex (ACC) in WIC.

The intensity of WIC experienced by an individual at any given moment can range from none, up to the maximum intensity associated with their Level of PD. As WIC is a dynamic state, it is suitable for study with resting-state functional connectivity (rsFC). (Biswal et al. 1995; Fox and Raichle 2007) In this experiment, we manipulated the intensity of WIC in order to study the effect on rsFC using the ACC as a seed for rsFC analysis.

The sensitization–homeostasis theory attributes the addictive potential of nicotine to inhibitory properties. (DiFranza and Wellman 2005; DiFranza et al. 2012a) According to the theory, neuroplastic changes develop to compensate for this inhibition, and during withdrawal, these homeostatic adaptations autonomously stimulate neural networks that generate WIC. The theory predicts that (1) there is a neural system involved in craving and that activity in this system correlates positively with the intensity of WIC; (2) activity in this system will be greater when smokers are in withdrawal than when they are satiated; and (3) since homeostatic changes stimulate this system during withdrawal, system activity in smokers during withdrawal will be greater than that of nonsmoking controls. By including nonsmoking controls, we were able to test and confirm these theory-driven predictions.

## Methods

### Subjects

The subjects were healthy smokers (*n* = 11) and nonsmokers (*n* = 10) of both genders recruited from the community via word of mouth and advertisements placed on the internet. Interested respondents were screened for eligibility by phone and then evaluated in person. Exclusionary criteria included a history of brain trauma, neurological conditions, substance abuse disorder, mental illness, use of psychotropic medications, and any contraindication to scanning. Subjects had to be between 18 and 39 years of age to reduce the likelihood of cerebrovascular damage from smoking. Nonsmokers must not have smoked more than two cigarettes, and none in the prior year. Smokers must have smoked daily for the past year and have a lifetime history of smoking >100 cigarettes. There was no minimum daily cigarette consumption required. The study was approved by the Committee for the Protection of Human Subjects at the University of Massachusetts Medical School.

### Behavioral measures

At the intake assessment, subjects completed a survey that collected demographic information and smoking history. The evaluation included three measures of nicotine addiction, a validated three-item instrument assessing the Level of Physical Dependence (PD), (DiFranza et al. 2011, 2012b,c) the Hooked on Nicotine Checklist (HONC), and the Fagerström Test for Nicotine Dependence (FTND). (Heatherton et al. 1991; Wellman et al. 2008) Nicotine withdrawal symptoms include craving, anger, irritability, frustration, anxiety, difficulty concentrating, restlessness, depression, increased appetite, insomnia, and impatience. (Hughes 2007) Of these, only WIC is addressed by the theory being tested. Smokers rated the intensity of their desire for a cigarette on a 100-mm visual analog scale under abstinent and satiated conditions. The magnitude of WIC that was present during the abstinent condition was calculated by subtracting the craving score for the satiated condition from that for the abstinent condition.

### Procedures

Eligible subjects returned on a scheduled date for functional MRI (fMRI) imaging. Subjects were instructed to refrain from alcohol consumption for 24 h prior to the study, to get a good night's rest, to eat a normal breakfast, and to drink a caffeinated beverage if that was their routine. Smokers were instructed not to smoke after 11 PM the night before the study and were told that their compliance would be tested with a carbon monoxide measurement. Upon arrival at the imaging center, all smokers reported that they had not smoked, and their CO measurements were consistent with this. All subjects provided a urine sample which was tested for drugs of abuse. Subsequently, subjects underwent two imaging sessions (35 min each) separated by a 15-min break. Immediately after the first imaging session, smokers rated their desire for a cigarette. During the break, smokers were instructed to smoke until they were satisfied; nonsmokers chewed non-nicotine gum for 5 min. Subjects then completed the second imaging session. Following the second scan, smokers again rated their desire for a cigarette. For the first imaging session (abstinent or withdrawal condition) it had been approximately 11 h since the last cigarette for smokers, and for the second session (satiated condition) they had been satiated by smoking ad lib 20 min before the rsFC data collection began.

### Imaging protocol

All MRI experiments were performed on a 3.0 T Achieva whole-body MR scanner (Philips Medical Systems, Best, the Netherlands).

Anatomical images were acquired using a high-resolution 3D T1-weighted sequence (MPRAGE) with the following parameters: TR/TE/flip angle of 7.4 ms/3.4 ms/8°, field of view (FOV) of 256 × 256 × 220, voxel size 0.98 × 0.98 × 0.6 mm. After the anatomical imaging, two fMRI images were acquired at resting state, separated by 15 min during which smokers smoked and nonsmokers chewed gum. Each fMRI lasted 7.5 min with the eyes closed using a single-shot gradient EPI sequence (TR = 1500 ms, TE = 35 ms, FOV = 230 × 230 ×120 mm, flip angle = 80°), 24 contiguous oblique-axial slices (2.7 × 2.7 × 4 mm voxels) parallel to the AC-PC line were obtained. DTI volumes were acquired and results are reported elsewhere. (Huang et al. 2013).

### Data preprocessing

Resting-state functional connectivity imaging data were preprocessed using Data Processing Assistant for Resting-State fMRI (DPARSFA; Chao-Gan and Yu-Feng 2010; http://www.restfmri.net) based on Statistical Parametric Mapping (SPM8) software (http://www.fil.ion.ucl.ac.uk/spm). The fMRI images were corrected for the acquisition delay between slices by shifting the signal measured in each slice relative to the acquisition of the slice acquired in the starting time of each TR. The head motion was corrected by estimating the values for translation (Hong et al. 2009;.) and rotation (degree) for each subject. Only subjects with head motion less than 2 mm in the *x*, *y* or *z* direction and less than 2° rotation about each axis were included. The motion corrected rsFC imaging volumes were spatially normalized to the standard SPM8 EPI template and resampled to 3 × 3 × 3 mm^3^. The processed images were then spatially smoothed with a 4 mm full width at half maximum (FWHM) Gaussian kernel. Linear trend removal and temporal band-pass filtering (0.01–0.08 Hz) were performed on the time series of each voxel. The individual T1-weighted MPRAGE images were coregistered to a standard stereotaxic space (MNI space; Brett et al. 2002) to facilitate group analysis.

Independent component analysis (Calhoun et al. 2001) was performed to evaluate changes in brain resting-state networks using GIFT toolbox (http://mialab.mrn.org/software/gift/index.html). Number of components was set to 20. The infomax algorithm was used to perform spatial ICA and spatial-temporal regression was chosen for back reconstruction. Independent components were scaled to *z*-scores (Calhoun et al. 2009). Resulted components were carefully compared to known resting-state networks that have been found consistently co-active during resting state (Damoiseaux et al. 2006; De Luca et al. 2006; van den Heuvel and Hulshoff Pol 2010; Allen et al. 2011). After the components were identified, the default mode network (DMN) was further examined for group comparisons.

In order to perform seed-based functional connectivity analysis, nuisance covariates (six head motion parameters, white matter and CSF signal) were removed from the aforementioned preprocessed images using linear regression within DPARSFA. Seed-based functional connectivity was analyzed using correlational analysis on a voxel-by-voxel basis. All Region of Interest (ROI) definitions were extracted using MarsBaR toolbox built in SPM8. rsFC maps for each seed of individual subjects were calculated using Resting-State fMRI Data Analysis Toolkit (REST (Song et al. 2011), http://www.restfmri.net). A regionally averaged time course from all voxels inside the seed region was used as a reference time course. For each subject, Pearson cross-correlation coefficients were computed between reference time courses and the time course of each individual voxel. Correlation coefficients were transformed using Fisher's *z* transformation to improve the Gaussian distribution.

### Analytic strategy

As previously reported, the DTI data from this study revealed that FA was higher in smokers than nonsmokers in the white matter of the ACb, and that FA in this area correlated with the level of PD (*r* = −0.68) and the HONC (*r* = −0.65). (Huang et al. 2013) For this reason, and because the ACC has been implicated in drug craving, (Brody et al. 2002, 2006; David et al. 2005; Lim et al. 2005; Wilson et al. 2005; Franklin et al. 2006; Rubinstein et al. 2010) we used the bilateral ACC as the seed for our rsFC analysis.

To examine rsFC under each condition (abstinent and satiated for smokers, before and after chewing gum for nonsmokers), we created rsFC maps for both groups for both conditions using one-sample *t* tests. In our protocol, the abstinence condition always preceded the satiated condition. To test for possible order effects and the stability of rsFC measurement in the absence of nicotine, we compared rsFC for the first and second scanning sessions for the nonsmokers using a paired *t* test. Next, to determine if nicotine withdrawal is associated with stronger ACC-seeded rsFC, we used a two-sample *t* test to compare rsFC maps for the abstinent condition for the smokers to that of the before gum chewing condition for the nonsmokers (the first of the two imaging sessions for both smokers and nonsmokers). To determine if ACC-seeded rsFC is stronger during nicotine withdrawal than nicotine satiation, we used a paired *t* test to compare the abstinent and satiated conditions for smokers. The rsFC map contrast of the abstinent condition versus the satiated condition identified areas where rsFC was different 11 h into withdrawal. As craving is only one of many symptoms triggered by nicotine withdrawal (irritability, impatience, difficulty concentrating, restlessness, etc. [Hughes 2007 #68]), it cannot be assumed that all neural activity that is augmented by withdrawal relates to WIC. To determine if there is a network of structures for which neural activity correlates with the intensity of WIC we conducted a correlation analysis to identify areas where the strength of rsFC (abstinent condition vs. satiated condition) correlated with the strength of WIC (craving score for the abstinent condition minus that for the satiated condition).

### Statistical analysis

Statistical analyses were done using SPM8, with threshold levels for significant differences set at *P* < 0.001, uncorrected at a voxel level, and *P* < 0.05, uncorrected for multiple comparisons at a cluster level.

## Results

Table 1 lists demographics and measures of nicotine dependence for smokers and nonsmokers. There was no significant age difference between smokers and nonsmokers. A comparison of rsFC between the first and second imaging sessions for the nonsmokers revealed no significant differences, indicating the stability of measurement and an absence of any order effects (Table 2A).

**Table 1:** Demographics and measures of nicotine dependence

	Smokers (*n* = 11)		Nonsmokers (*n* = 10)	
		
Subjects	Mean	SD	Mean	SD
Age (years)	23.7	1.98	22.5	6.78
FTND	4.0	1.69	–	–
HONC	6.0	2.88	–	–
Level of PD	1.6	0.92	–	–

HONC, Hooked on Nicotine Checklist; PD, physical dependence.

**Table 2:** Summary of results showing peak clusters

	Coordinates			
		
Results	*x*	*y*	*z*	Peak *P* or *r*
A. No significant difference was found between the ACC-seeded rsFC maps in nonsmokers for the first and second imaging sessions.				
B. Eleven hours into withdrawal, smokers showed stronger ACC-seeded rsFC than nonsmokers to the…				
L precuneus	−18	−63	39	
R precuneus	31	−66	39	
L middle temporal gyrus	−48	−30	−6	
R middle frontal gyrus	33	45	12	
L middle frontal gyrus	−36	45	9	*P* < 0.05
R putamen	19	0	8	
R caudate	10	0	13	
L inferior temporal	−52	−48	−16	
L inferior parietal lobe	−42	−48	24	
C. Compared to the abstinent condition, smokers in withdrawal showed stronger ACC-seeded rsFC to the…				
R anterior cingulate gyrus	3	39	0	
R precuneus	3	−42	45	
L precuneus	−6	−84	39	
L insula	−38	−15	13	*P* < 0.02
R insula	42	−18	12	
L inferior orbital frontal gyrus	−41	36	−16	
R inferior orbital frontal gyrus	42	37	−16	
D. The strength of Withdrawal-Induced Craving correlated with rsFC in the…				
L precuneus	−11	−65	17	*r* = 0.64
R insula	34	25	6	*r* = 0.76
L insula	−43	3	7	*r* = 0.72
R middle cingulate gyrus	3	−3	37	*r* = 0.68
R precentral gyrus	51	−7	11	*r* = 0.60
L postcentral gyrus	−55	−15	13	*r* = 0.69
L putamen	−12	9	−9	*r* = 0.72
L caudate	−12	14	6	*r* = 0.62

ACC, anterior cingulate cortex; rsFC, resting-state functional connectivity.

**Figure 1:**
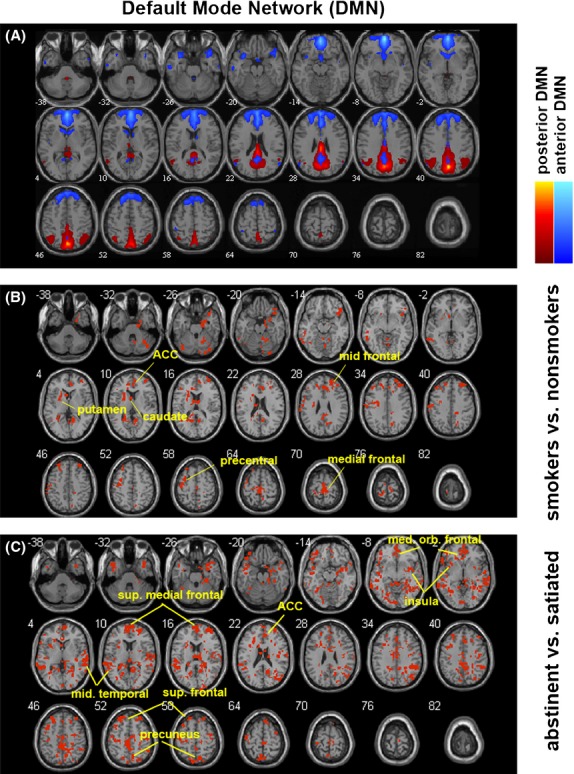
Results from independent component analysis (ICA), particularly in the default mode network (DMN). (A) Components that formed the DMN, including the posterior DMN and the anterior DMN. (B) Difference within the DMN between nonsmokers and smokers during abstinence, shown in binary mask. Compared to nonsmokers, smokers in the abstinent condition showed enhanced connectivity in their DMN to areas of the anterior cingulate cortex (ACC), caudate, putamen, middle frontal area, precentral gyrus, and the medial frontal gyrus. (C) Difference in smokers' DMN between abstinent (withdrawal) and satiated conditions. When compared to the satiated condition, DMN of smokers in the abstinent condition had enhanced connectivity to areas of the ACC, precuneus, medial orbital frontal area, insula, superior medial frontal area, middle temporal gyrus, and superior frontal area. Red binary masks in B and C indicate differences at a significance level of *P* < 0.05.

After excluding components of noise and motion, 13 components were identified from ICA output corresponding to the following networks: cerebellum-hippocampal-precuneus, inferior frontal gyrus-mid temporal, posterior DMN, motor, visual (two), right executive, anterior DMN, supplementary motor, auditory, left executive, parietal, and salience network. The DMN, comprising the anterior and posterior DMN (Fig. 1A) was further examined for group comparisons. Compared to nonsmokers, a two-sample *t* test showed enhanced connectivity in the DMN of smokers in the abstinent condition to areas of ACC, caudate, putamen, middle frontal area, precentral gyrus, and the medial frontal gyrus (Fig. 1B). When compared to the satiated condition, DMN of smokers in the abstinent condition had enhanced connectivity to areas of the ACC, precuneus, medial orbital frontal area, insula, superior medial frontal area, middle temporal gyrus, and superior frontal area (Fig. 1C).

**Figure 2:**
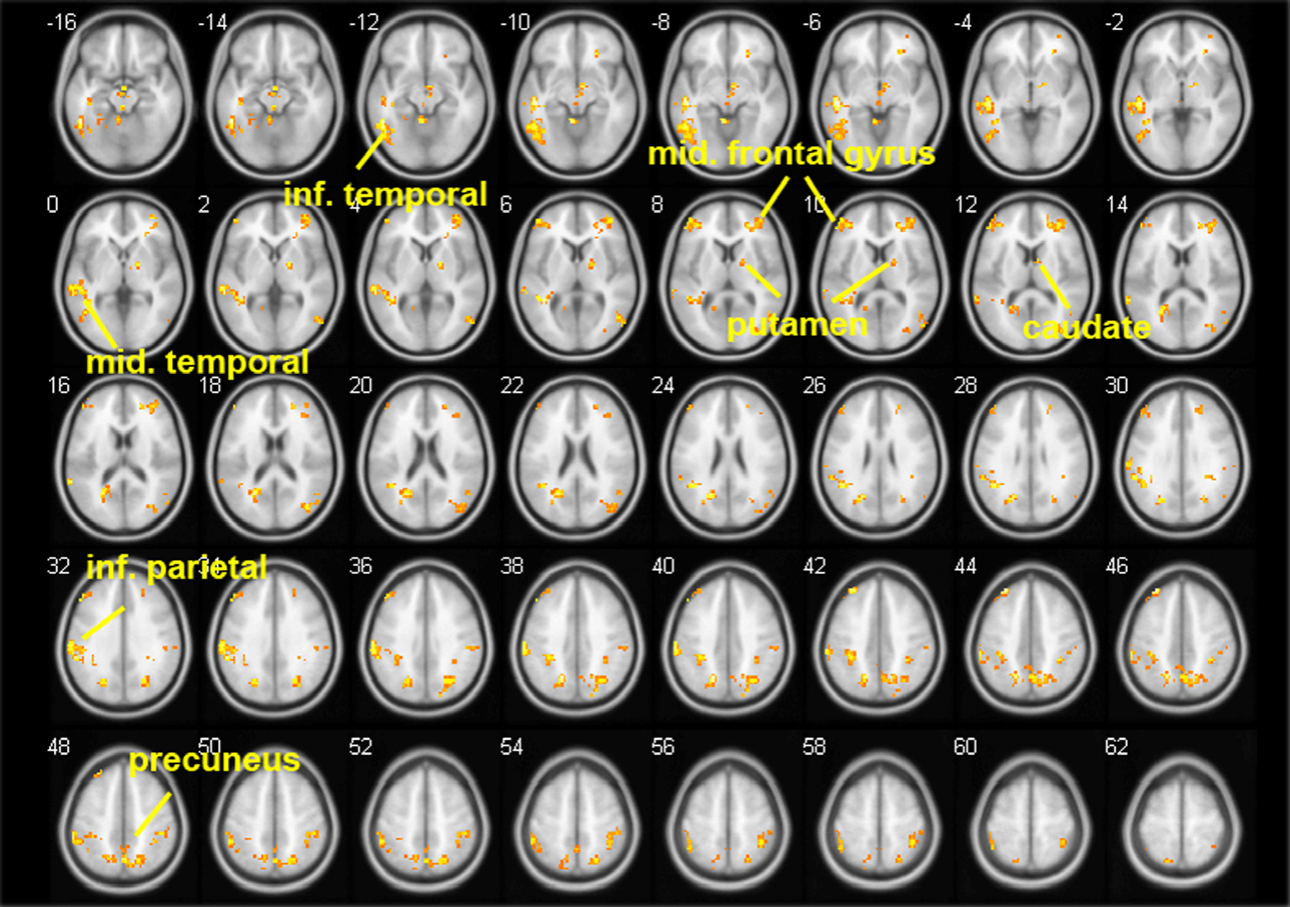
Comparing anterior cingulate cortex (ACC)-seeded resting-state functional connectivity (rsFC) in smokers during the abstinent condition to that of nonsmokers. Smokers showed greater rsFC in the precuneus, caudate, putamen, superior frontal gyrus, middle frontal gyrus, superior parietal gyrus, superior occipital lobe, inferior parietal lobe, middle temporal lobe, and the inferior temporal gyrus (*P* < 0.05). There were no areas showing decreased rsFC in smokers in the withdrawal condition as compared to controls. Brighter color indicates a higher *t* value.

**Figure 3:**
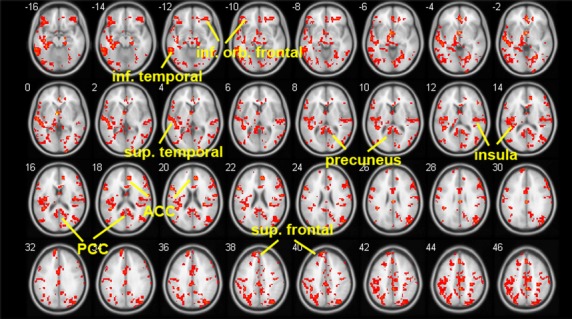
Areas of increased resting-state functional connectivity (rsFC) during withdrawal. To identify changes in rsFC associated with nicotine withdrawal, paired *t* test was implemented between the anterior cingulate cortex (ACC)-seeded rsFC maps for the abstinent condition and that for the satiated condition. Areas that showed stronger rsFC during withdrawal (shown in red) compared to satiated condition included: the precuneus, insula, orbital frontal gyrus, superior frontal gyrus, posterior cingulate cortex, superior temporal lobe, and the inferior temporal lobe (*P* < 0.02). PCC-posterior cingulate cortex.

**Figure 4:**
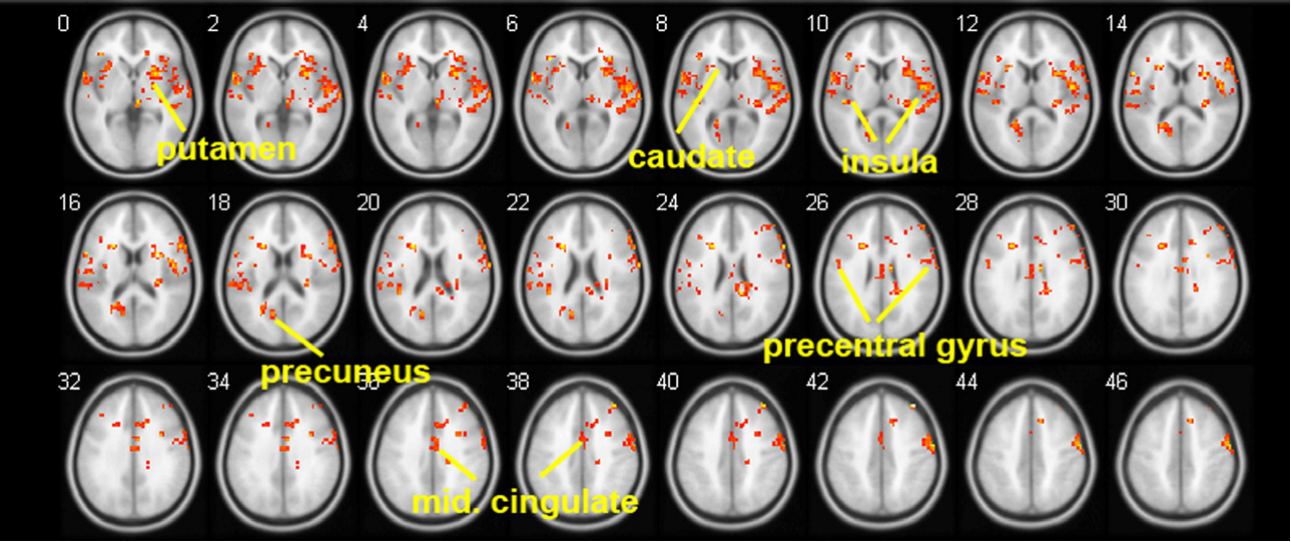
Areas where resting-state functional connectivity (rsFC) in the abstinent state correlates with the intensity of Withdrawal-Induced Craving. Highly correlated areas (shown in red) included: the precuneus, insula, caudate, putamen, middle cingulate gyrus, and precentral gyrus (*P* < 0.05). The peak correlation in the insula was *r* = 0.76.

Smokers in the abstinent state showed stronger ACC-seeded rsFC than nonsmoking controls in the precuneus, caudate, putamen, frontal cortex, temporal cortex, and inferior parietal lobe (*P* < 0.05, Table 2B, and Fig. 2). The comparison of smokers in the satiated and abstinent conditions revealed that withdrawal from nicotine for 11 h was associated with increased rsFC between the ACC and the precuneus, insula, orbital frontal gyrus, superior frontal gyrus, posterior cingulate cortex, superior temporal lobe, and the inferior temporal lobe (*P* < 0.02, Table 2C, Fig. 3). WIC significantly correlated (peak *r* = 0.76) with the strength of rsFC in the precuneus, insula, caudate, putamen, middle cingulate gyrus, and precentral gyrus (*P* < 0.05 Table 2D, Fig. 4). Table 2D summarizes the results with the corresponding peak clusters.

## Discussion

Addiction researchers have long hypothesized that PD is attributable to the development of neural adaptations. By employing a new survey measure of PD, (DiFranza et al. 2011) we were able to demonstrate that the progressive development of PD is associated with decreasing FA in the ACb (*r* = −0.68), increasing density of white matter tracts between the ACb and white matter approaching the precuneus (*r* = 0.75), and decreasing density of white matter tracts between the ACb and the frontal lobe (*r* = −0.86). (Huang et al. 2013) The density of tracts between the ACb and white matter approaching the precuneus also correlated with scores on the HONC and the FTND. (Huang et al. 2013) These observations suggested that neural adaptations in the ACC-precuneus circuit might play a key role in the development of PD.

Despite the small sample size, our results using two different methods (ICA and ACC-seed based rsFC analysis) were highly consistent. We found that 11 h into withdrawal, abstinent smokers showed increased rsFC in many pathways as compared to the satiated condition (Table 2B), and that rsFC in smokers in withdrawal was greater than that of nonsmoking controls. Our analysis identified several pathways connecting to the ACC in which rsFC was significantly correlated with the intensity of WIC (Table 2D, Fig. 4). These included pathways involving the precuneus, insula, caudate, putamen, middle cingulate gyrus, right precentral gyrus, and left post central gyrus. While we studied correlates of withdrawal-induced craving in the absence of cues, others have studied correlates of cue-induced craving. Cue-induced craving has been shown to correlate with activity in the ACC, precuneus, precentral gyrus, and postcentral gyrus. (McClernon et al. 2005; Culbertson et al. 2011) Our results suggest that there is considerable overlap in the brain regions that are involved in cue-induced and withdrawal-induced craving. This supports the sensitization–homeostasis theory's prediction that both smoking cues and withdrawal would activate a common craving pathway. (DiFranza and Wellman 2005; DiFranza et al. 2012a).

The ACC and precuneus are both major components of the DMN. (Ding and Lee 2013) Prior studies suggest that nicotine suppresses activity in the DMN, while nicotine withdrawal appears to activate it. (Cole et al. 2010; Sutherland et al. 2012) In the only prior rsFC study of WIC, WIC correlated with increased rsFC between the precuneus and the default mode network. (Cole et al. 2010) Ding and Lee found increased rsFC in the abstinent state in circuits connecting the ACC, precuneus and insula, however, they did not measure WIC. (Ding and Lee 2013) Thus, three studies have now shown that nicotine withdrawal is associated with increased rsFC in the precuneus, and in two studies, rsFC in precuneus circuits correlated with the severity of WIC.

The involvement of the ACC-precuneus pathway in craving is consistent with prior research. ACC activation has been linked to smoking cue reactivity (Brody et al. 2002; Lim et al. 2005; McClernon et al. 2005, 2009; Franklin et al. 2006; Culbertson et al. 2011; Li et al. 2013) and nicotine craving. (Daglish et al. 2001; Brody et al. 2002, 2006; David et al. 2005; Lim et al. 2005; Wilson et al. 2005; Franklin et al. 2006; Rubinstein et al. 2010; Li et al. 2013) A recent meta-analysis found a reliable smoking cue reactivity effect in the precuneus. (Hartwell et al. 2011; Engelmann et al. 2012) Lower glutamate levels in the dorsal ACC have been associated with increased risk of early relapse during smoking cessation. (Mashhoon et al. 2011) Using real-time biofeedback, investigators demonstrated that volitional reduction in ACC activity was associated with a reduction in craving for tobacco. (Li et al. 2013) Active resistance to cue-induced craving in bupropion-treated smokers was associated with reduced activation in the precuneus and ACC. (Culbertson et al. 2011).

We found that WIC correlated with increased rsFC in smokers in the ACC-caudate and ACC-putamen circuits. Hong et al. (2009) found that a genetic variant of the nicotinic receptor was associated with rsFC between the ACC and striatum which correlated with the FTND. (Hong et al. 2010) Gloria et al. reported activation in the ACC and caudate in anticipation of a nicotine infusion in abstinent smokers. (Gloria et al. 2009).

Naqvi et al. reported that stroke lesions to the insula increase the likelihood of quitting smoking, suggesting that the insula is a critical neural substrate in tobacco addiction. (Naqvi et al. 2007) A role for the insula in tobacco addiction is also supported by our fMRI finding that rsFC between the insula and ACC increases during withdrawal. Sutherland et al. propose that “during nicotine abstinence, the insula may track withdrawal-induced bodily sensation and in turn direct attention towards this homeostatically salient internal state via increased interaction with the default mode network at the expense of decreased exogenously directed attention mediated by the executive control network.”(Sutherland et al. 2012) In their model, the insula works in conjunction with the ACC. This model fits nicely with the sensitization–homeostasis theory, which addresses how nicotine withdrawal generates a “homeostatically salient internal state.” Our data provide some support for the Sutherland model by demonstrating that the withdrawal condition is associated with increased rsFC between the ACC and the insula that correlates with the intensity of WIC (Table 2C and D).

Our structural analysis revealed a strong negative correlation between PD and white matter tracts connecting the frontal cortex and ACC. We speculate that this might be related to a loss of “top down” control of executive function over craving networks. (Heatherton and Wagner 2011) In a biofeedback experiment, smokers were unable to “increase resistance” to tobacco craving by increasing activity in the medial prefrontal cortex. (Li et al. 2013) We observed stronger rsFC between the ACC and the frontal cortex during abstinence than during satiation, and in abstinent smokers as compared to nonsmoking controls. However, this activity did not correlate with WIC. ACC-frontal lobe circuits might relate to the brain's attempts to suppress craving or to cope with disruptions to homeostasis caused by withdrawal, as suggested by Sutherland et al. (Sutherland et al. 2012).

Strengths of our study design include a theory-driven analysis, the recruitment of smokers that represented the full spectrum of PD which allowed us to identify structural changes that track the progression of PD, the combination of structural and functional analyses, standardization of the length of abstinence for smokers, and the inclusion of nonsmoking controls.

A limitation of this study was the potential for order effects given that the abstinence condition always preceded the satiated condition. This could have been avoided by randomizing half the smokers to complete the satiated condition first, but this would have required 2 days of imaging for each subject, which was not possible given our budgetary limitations. We used a measure of WIC rather than a standardized instrument that addresses all withdrawal symptoms because of uncertainty about whether withdrawal symptoms such as headache, nausea, and increased appetite are central nervous system symptoms.

Another limitation of this study is the small sample size. The small sample size made it relatively difficult to identify brain networks from the ICA results, thus further validation of our findings is necessary. However, the two approaches used in this study (ICA and seed-based analysis) presented highly consistent results, which suggest the robustness of our findings.

In summary, (1) increasing density of neural tracts between the ACC and precuneus correlates (*r* = 0.75) with the progressive development of PD (Huang et al. 2013); (2) compared to the satiated state, rsFC in this pathway (and several others) increases when smokers are in withdrawal; (3) rsFC in this pathway (and several others) is stronger in smokers in withdrawal than in nonsmoking controls; and (4) during withdrawal rsFC in this pathway (and several others) correlates with the intensity of WIC (*r* = 0.64). These data suggest that the development of nicotine addiction may be associated with the development of neural adaptations that support the experience of craving during nicotine withdrawal. These results are consistent with the predictions of the sensitization–homeostasis theory (8, 9).
